# A Novel Biocidal Nanocomposite: Spherical Silica with Silver Ions Anchored at the Surface

**DOI:** 10.3390/ijms24010545

**Published:** 2022-12-29

**Authors:** Magdalena Laskowska, Paweł Kowalczyk, Agnieszka Karczmarska, Karol Kramkowski, Karol Wrzosek, Łukasz Laskowski

**Affiliations:** 1 Institute of Nuclear Physics Polish Academy of Sciences, PL-31342 Krakow, Poland; 2 Department of Animal Nutrition, The Kielanowski Institute of Animal Physiology and Nutrition, Polish Academy of Sciences, PL-05110 Jabłonna, Poland; 3 Department of Physical Chemistry, Medical University of Bialystok, PL-15089 Białystok, Poland; 4 Department of Heart Diseases, The Medical Center of Postgraduate Education, PL-01813 Warszawa, Poland

**Keywords:** spherical silica, functional materials, nanocomposites, Fpg glycosylase, oxidative stress, bacterial *E. coli* strains, antibiotics, LPS

## Abstract

This article is devoted to a novel class of antimicrobial agents: nanocomposites composed of spherical silica and silver ions located at the silica’s surface with the assumed distribution. Such materials are in high demand due to the increasing threat from bacterial strains that are becoming resistant to currently known antibiotics. In particular, we focus on materials that make it possible to limit the growth of bacterial colonies on a variety of tactile surfaces. In this paper, we present a method for preparing a silica-based nanocomposite containing silver ions and the analysis of their antimicrobial properties. Our research revealed that the presence of tested nanocomposite induces very high oxidative stress in the bacteria cell, damaging and modifying bacterial DNA, creating oxidized guanines, cytosines, or adenines, which causes its very rapid destruction, leading to cell death.

## 1. Introduction

Recently, a substantial increase in using antimicrobial nanomaterials has been noticed, mainly due to the COVID-19 pandemic (infection of SARS-CoV-2). Metals, such as silver or copper, can be toxic to bacteria or viruses, having antimicrobial activities at exceptionally low concentrations. Moreover, unlike other antimicrobial agents, they are stable under conditions currently found in the industry, allowing for their use as additives. Especially copper and silver-based compounds have been widely used as antimicrobial agents [1] in a multitude of applications in agriculture, healthcare, and general industry. Using metallic copper or silver in industrial applications involves several challenges associated with the nature of the metal itself. Silver is relatively expensive, while copper is susceptible to the corrosion process. Nevertheless, metal nanoparticles have become more popular.

Most of the studies found in the literature on the antimicrobial activities of metal nanostructures are restricted to strains of pathogenic microorganisms and rarely extend to non-pathogenic eco-friendly microorganisms [2,3,4,5]. Hence, the impacts of the environmental accumulation of nanoparticles, especially on microbial communities residing in inland fields or bodies of water, which are critical to environmental recycling, are unknown. For this reason, preventing the release of metallic nanoparticles into the environment and their absorption by living organisms seems to be a vital task.

As has been proven, the nanoparticle form of silver or copper exhibits strong antimicrobial properties [6,7,8], which increase with the decrease in the hydrodynamic size [9,10,11]. Therefore many research teams are struggling to obtain the smallest metal nanoparticles. As can be easily concluded, the limiting size of such nanoparticles would be a single atom. How can we, however, separate individual atoms?

**Figure 1:**
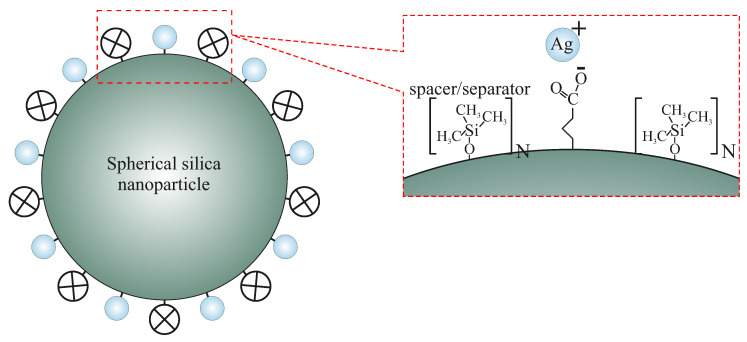
The scheme of spherical silica functionalized by silver ions with designed inter-molecular distances. The spacer units play the role of separators between silver-containing functional units. N denotes the statistical number of spacer units between functionalities.

In this article, we propose the material that can solve the above-mentioned problems. We present the composite material composed of spherical silica (250 nm in diameter) matrix with silver ions homogeneously distributed at its surface. Moreover, the silver ions’ distribution can be finely tuned by using the 2D solid solvent approach [12,13], as depicted in Figure 1. In this case, the density of functional units can be modified by a variation in proportions between anchoring and spacer units.

By the use of silica matrix, the composite material can be easily used as polymers, glass ionomers, or cement fillers [14,15,16,17] in all kinds of applications where pathogens elimination is crucial [18,19]. This strongly reduced possibility of metal migration outside the active area. What is more, biocidal action is local and with significantly limited antimicrobial agent depletion (active molecules anchored to silica surface).

In this article, we investigate the above-mentioned material containing three concentrations of silver ions at the surface. The concentrations were set by the number of spacer units: the higher the number of spacer units per single functional molecule, the less frequent distribution of functionalities. For this investigation, we choose 1, 6, and 12 spacers per functional unit. Thus, we named samples consequently:

- *SilS-COOAg 1*—spherical silica functionalized with silver ions, containing one spacer unit per single functional group (the highest concentration of silver among all the samples).
- *SilS-COOAg 6*—spherical silica functionalized with silver ions, containing six spacer units per single functional group.
- *SilS-COOAg 12*—spherical silica functionalized with silver ions, containing 12 spacer units per single functional group (the lowest concentration of silver among all functionalized samples).

Samples were analyzed with regard to their antimicrobial properties and dependencies of biocidal action from the distribution of the silver ions. Additionally, we tested pristine spherical silica as a reference (the sample named *RefSilS*).

## 2. Results and Discussion

### 2.1. Transmission Electron Microscopy Imaging

**Figure 2:**
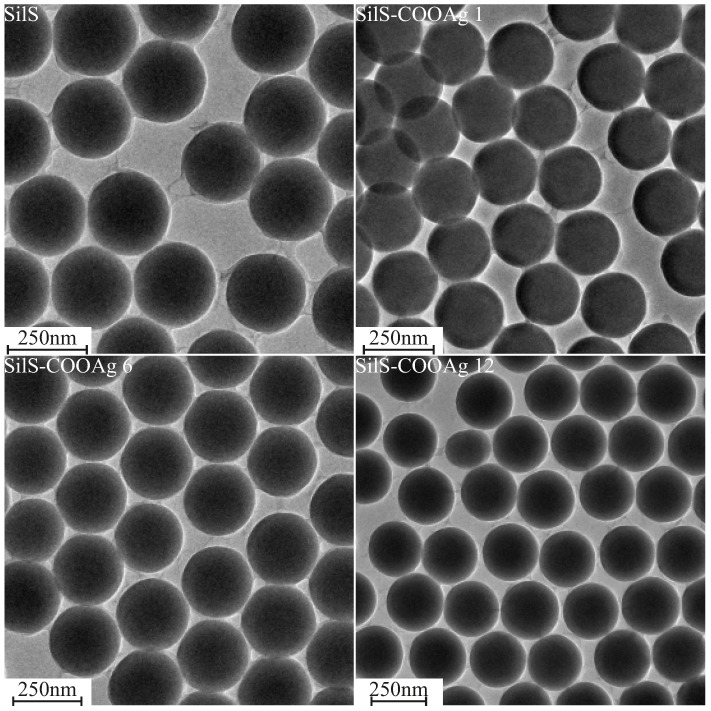
TEM images of functionalized samples (SilS-COOAg 1, SilS-COOAg 6, and SilS-COAg 12) and reference—pure spherical silica (SilS).

As the first evidence of the synthesis results, we carried out transmission electron microscopy. TEM observations allowed for checking the purity of samples, excluding the possibility of silver agglomeration at the silica’s surface. The obtained microphotography of the investigated samples juxtaposed with pristine spherical silica as a reference, can be seen in Figure 2.

The reference material—spherical silica before functionalization—shows the correct structure: the spheres are homogeneous with a diameter of 250 nm, according to our assumption (Figure 2—upper row). Neither impurities nor structural defects are visible. For this reason, we can assume, that synthesis was started from the correct substrate material.

After functionalization, the structure did not visibly change (Figure 2—pictures labeled SilS-COOAg 1, SilS-COOAg 6, and SilS-COAg 12), i.e., the shape and diameter of the spheres are unaltered. Neither impurities can be seen nor agglomeration of particles. On this basis, we can conclude that in the case of functionalized material, only individual molecules are attached to the surface instead of bulk material, which would be clearly visible under TEM.

### 2.2. Antimicrobial Tests

As-synthesized materials were undergone antimicrobial tests.

**Figure 3:**
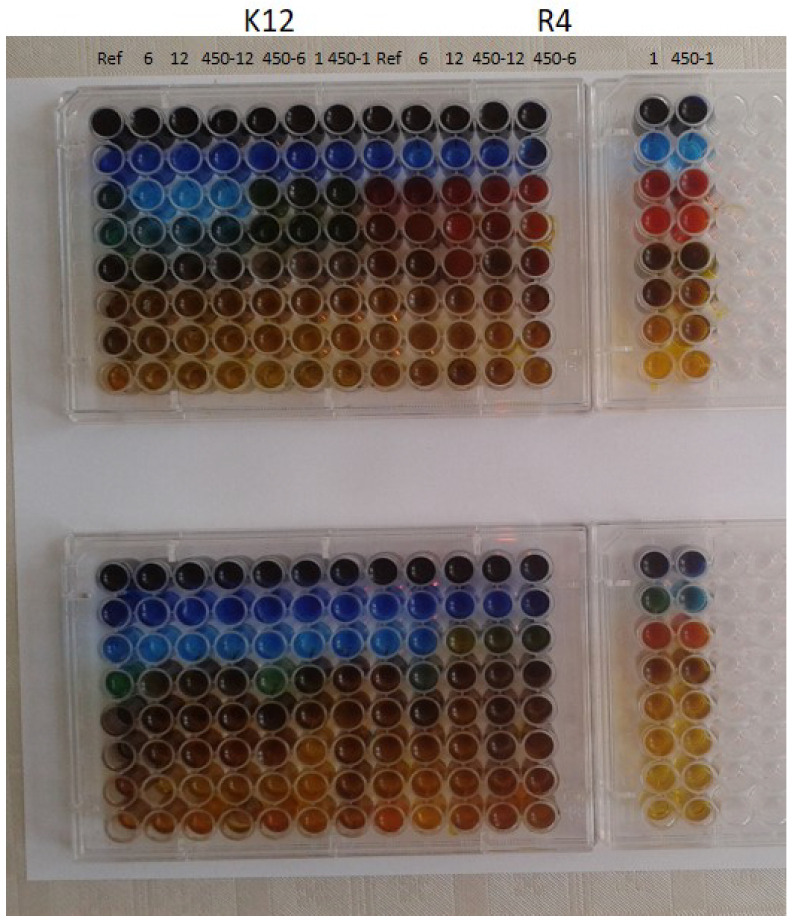
Examples of MIC and MBC on microplates with different concentrations of studied compounds (μg/mL${}^{-1}$). The numbers correspond to sample names: 1 denotes SilS-COOAg1, 6 corresponds to SilS-COOAg6, and 12 to SilS-COOAg 12. RefSilS was marked as Ref. All experiments were performed in duplicate. Resazurin was added as an indicator of microbial growth with K12, R4 strains with tested Ag matrices.

The model bacterial strains of *E. coli* K12, R2-R4, were treated with the analyzed spherical silica with silver ions using MIC and MBC assays with 48-well plates. Color changes were observed for all tested compounds but at different levels and at different dilutions. The most susceptible to modification with these compounds were the bacterial strains R4 due to the increased length of their lipopolysaccharides (LPS, visible dilutions 10${}^{-2}$ corresponding to the concentration of 0.0015 μM) in comparison to strains K12 and R2 (visible dilutions 10${}^{-6}$ corresponding to the concentration of 0.0015 μM and 0.02 μM for K12 and R2, respectively, as can be seen in Figure 3.

**Figure 4:**
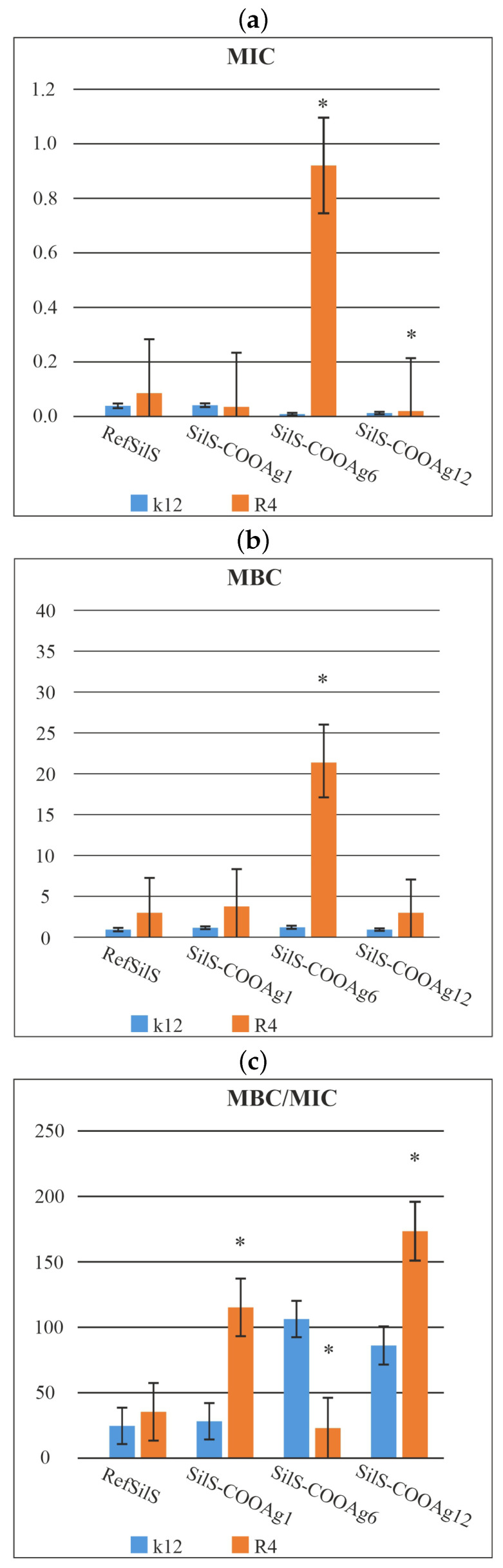
Minimum inhibitory concentration (MIC—**a**), minimum bactericidal concentration (MBC—**b**), and the ratio of MBC/MIC (**c**) of the SilS in model bacterial strains. The x-axis features compounds RefSilS, SilS-COOAg1, SilS-COOAg6, SilS-COOAg12 used sequentially. The y-axis shows the MIC value in μg/mL${}^{-1}$. Investigated strains of *E. coli* K12 as control (blue), and R4 strain (orange). The sign * means statistical significance below 0.05.

The analyzed R4 strain was the most sensitive of all strains, most likely due to the longest length of the lipopolysaccharide chain in the bacterial membrane, which may curl relaxing depending on the used material. In all analyzed cases, the MBC values were approximately 260 times higher than the MIC values (Figure 4). Based on the observed results in the MIC and MBC tests, it was found that the analyzed spherical silica functionalized by silver ions significantly influenced the fragmentation of the membrane and the structure of the cell wall of bacteria containing LPS of various lengths [20,21,22,23,24,25]. This causes high oxidative stress in the cell which affects the damage and modification of bacterial DNA caused by the analyzed nanocomposites. This was additionally confirmed by the digestion with the specific enzyme Fpg of modified bacterial DNA (Labjot, New England Biolabs, UK), which is a marker of oxidative stress [20,21,22,23,24,25,26] recognizing oxidized guanine and adenine (Figure 4). The highest values after the application of the membranes in model strains of *E. coli* after the use of MIC, MBC, and MBC/MIC tests were observed after treatment with selected silver-containing nanocomposites. They were increased in all strains, more in R4, and comparable with each other after the use of analyzed spherical silica with silver ions. The highest value was observed for the R4 strain, whose LPS length was the longest and was relaxed, inducing significant oxidative stress in the cell.

Compounds of plant origin, bacteriophages, and their lytic enzymes or the latest nanotechnology products, biogenic nanosilver or gold, copper, and platinum nanoparticles have been used against selected microorganisms, such as multidrug-resistant bacteria (MDRB) [27]. Promising new developments in nanotechnology make it possible to combat microbes. There are several technologies for the synthesis of NPs and nanocomposites (e.g., peptidomimetics) with strictly defined physicochemical properties and significant biological potential [28,29,30]. It allows direct access of the nano-drug to a specific organ, which reduces side effects and facilitates its delivery to cells. The effect of silver concentration in the applied nanocomposites with different concentrations from 1, 6, and 12 with three different proportions of BNTES and TEOS which correspond to the materials: SilS-COOAg 1, SilS-COOAg 6 and SilS-COOAg 12 are very similar in their action to that of nanosilver particles. Nano-silver particles inhibit the growth and development of gram-negative bacteria (e.g., Escherichia, Pseudomonas) and gram-positive bacteria resistant to antibiotics (Bacillus, Staphylococcus, and Streptococcus). Nanoparticles in combination with some antibiotics may increase their effectiveness [31,32,33,34]. The bactericidal effect of silver is related to the mechanism of interaction of thiol groups with the bacterial cell wall, increasing the permeability of the cell membrane, causing ionic disturbances, and the destruction of bacterial DNA. Under the influence of silver, metabolic products accumulate in the cell, and the possibility of synthesizing their proteins and sugars is stopped [32,33,34], which leads to the death of the bacterial cell. The mechanisms of cell death induction by AgNPs are particularly observed in model *E. coli* bacteria, especially in strains R2–R4 containing LPS of various lengths. In addition, AgNPs, similar to the various types of peptidomimetics used, can destroy the bacterial membrane’s structures by increasing its permeability and creating many micropores that penetrate into the bacterial cell, inducing oxidative stress [33,34]. During this process, the DNA structure is broken down by oxidation and alkylation of the bases, which leads to numerous modifications of the DNA bases, such as 8-oxoguanine, 7,8-dihydro-8-oxoguanine (8-oxoguanine), 8-oxoadenine-unsubstituted and substituted, ring-opened imidazole purines introduced into DNA by hydroxyl radicals (e.g., FapyG, FapyA), as well as chemical carcinogens, including anticancer drugs (e.g., Fapy-7MeG, Fapy-7EtG, Fapy-7aminoethylG, aflatoxin B1-fapy-guanine, 5-hydroxycytosine and 5-hydroxyuracil), fapy-adenine (FapyG) and fapy-guanine (FapyA) [20,35]. So far, bacteria have not been able to develop effective defense mechanisms against AgNPs, and against other metals such as gold, copper, and platinum against pathogens bacterial strains present in nosocomial infections, which include *S. aureus*, *S. mutans*, *E. coli*, *P. aeurginosa*, strains of Salmonella and Campylobacter [36,37,38,39,40,41,42,43]. The same effect is also observed in the applied nanocomposites in model *E. coli* strains.

## 3. Materials and Methods

### 3.1. Synthesis of Silica Nanoparticles Functionalized Silver Ions

First, the monodisperse 250 nm spherical silica nanoparticles were prepared according to the optimized Stöber procedure [12,44,45]. During the synthesis, a solution containing 41.5 mL of ethanol (99%), 4 mL of ammonia (25%—Sigma-Aldrich Chemie GmbH, Schnelldorf, Germany), and 1.5 mL of distilled water (supplied from our laboratory) was carried out in a 100 mL round-bottom flask under a magnetic stirring (>900 rpm) at a temperature of 40 ${}^{{}^{\circ}}$C for 15 min. After this period, 3 mL of tetraethyl orthosilicate (TEOS—Sigma-Aldrich Chemie GmbH, Germany, purity 99%) was added to the mixture, and its hydrolysis and condensation were left to occur for 2 h at the same conditions. The final concentration of reagents in the solution was as follows: TEOS—0.27 mol/L, ammonia—0.52 mol/L, and water—4.68 mol/L. After the hydrolysis and polycondensation of TEOS, the obtained monodisperse SiO${}_{2}$ particles were purified by centrifugation (6000 rpm, 20 min) and washed three times with water and ethanol for removing reagents adsorbed on the surface of the particles. Finally, they were dried overnight at 80 ${}^{{}^{\circ}}$C before further processing.

**Figure 5:**
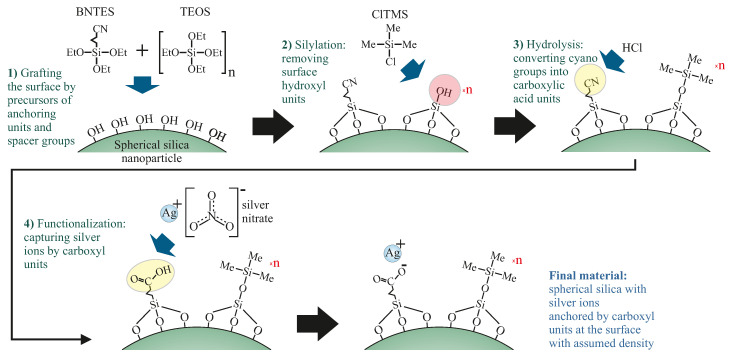
A diagram of the synthesis route of the spherical silica functionalized by silver ions anchored by propyl carboxylic acid groups with an assumed distribution. The number “n” denotes the number of tetraethyl orthosilicate (TEOS) in moles per single mole of the precursor of functional units (butyronitriletriethoxysilane—BNTES) and determines the density. Other abbreviations: TEOS—tetraethyl orthosilicate, ClTMS—chlorotrimethylsilane, Me—methyl groups, Et—ethyl groups.

Next, as-synthesized spherical silica was functionalized by silver ions with assumed density at the surface, according to the steps presented in Figure 5.

In the first step (**1**), the precursors of anchoring groups (butyronitriletriethoxysilane—BNTES—Sigma-Aldrich Chemie GmbH, Germany, purity 98%) and spacers (TEOS) were dissolved in dichloromethane (Sigma-Aldrich Chemie GmbH, Germany, purity 99.8%) with a total concentration of 10${}^{-1}$ mol/dm${}^{3}$. The molar proportion of BNTES and TEOS determines the distances between functional units: BNTES creates the anchoring unit for silver ions, while TEOS is a precursor of the spacer.

As mentioned before, we prepared materials with three different concentrations of functional units, so with three different ratios of BNTES and TEOS—1:1, 1:6, and 1:12—which correspond to the following materials: SilS-COOAg 1, SilS-COOAg 6, and SilS-COAg 12, respectively. Next, dry spherical silica was added and the suspension was rigorously mixed under reflux for 24 h. The pre-functionalized powder was recovered by centrifugation and washed several times with dichloromethane. After this procedure, the resulting powder was thoroughly dried in a vacuum at 120 ${}^{{}^{\circ}}$C for at least 24 h and stored in a protective atmosphere.

In the next step (**2**), the material was silylated in order to convert hydroxyl units into trimethyl silane groups (constituting spacers). To do this, Chlorotrimethylsilane (ClTMS—Sigma-Aldrich Chemie GmbH, Germany, purity 98%) was dissolved in dichloromethane with a total concentration of 5 × 10${}^{-2}$ mol/dm${}^{3}$. After obtaining a clear solution, we added a dry pre-functionalized silica powder (1 g per 50 mL of solution) and stirred it under reflux for 24 h. The silylated powder was recovered by centrifugation and washed several times with dichloromethane. Next, the material was dried in a vacuum at 120 ${}^{{}^{\circ}}$C for at least 24 h.

In the third step (**3**), we hydrolyzed the precursors of anchoring units into carboxylic acidic groups. The silica powder was dispersed in a mixture of concentrated HCl (concentration of 37%), acetone, and deionized water (2:1:1). The addition of acetone is necessary because the surface of silica spheres is strongly hydrophobic after silylation. We added 50 mL of acid to each gram of powder. The suspension was mixed under reflux for 24 h. The functionalized silica powder was recovered by centrifugation and washed several times with a mixture of deionized water and acetone until neutral pH was obtained. After drying in a vacuum at 120 ${}^{{}^{\circ}}$C for at least 24 h, the material was ready for functionalization.

The last step (**4**) was the functionalization of the anchoring units (carbonic acid) by silver ions. This procedure must be carried out in total darkness to avoid the crystallization of silver. To do this, we prepared the solution (10${}^{-3}$ mol/dm${}^{3}$) of AgNO${}_{3}$ (Sigma-Aldrich Chemie GmbH, Germany, purity ≥ 99%) in deionized water and acetone (1:1). The addition of acetone is necessary since pre-functionalized silica powder is strongly hydrophobic. We added 50 mL of such solution for each gram of pre-functionalized silica. The suspension was mixed under reflux for 24 h. Similarly to the previous steps, the powder was recovered by centrifugation and washed several times with a mixture of DI water and acetone. The resulting materials were dried in a vacuum for a few hours and stored under a protective atmosphere in dark bottles.

### 3.2. Characterization Methods

The sample morphologies were confirmed by transmission electron microscopy (TEM) with an FEI Tecnai G2 20 X-TWIN equipped with a LaB${}_{6}$ emission source and an FEI Eagle 2 K CCD camera. The samples for TEM observations were prepared by the protocol commonly used for nanoparticles, described in the literature [46].

### 3.3. Microorganisms and Media

*Escherichia coli* strains R1–R4 and K-12 were obtained as a kind gift from Prof. Jolanta Łukasiewicz at the Ludwik Hirszfeld Institute of Immunology and Experimental Therapy 86 (Polish Academy of Sciences), where the microorganisms were cultured. The reference bacterial strains of *E. coli* (K12 ATCC 25404, R2 ATCC 39544, R3 ATCC 11775, R4 ATCC 39543 were provided by LGC Standards (U.K.) and were used according to the recommendation of ISO 11133: 2014. These strains were used to test the antibacterial activity of the synthesized agents [20,21,22,23,35,47,48]. Bacteria were grown in liquid medium or agar plates containing tryptic soy broth medium (TSB, Sigma-Aldrich Saint Louis, MI, USA), using a methodology described previously [24,25,49,50].

### 3.4. Statistical Analysis

All obtained data were tested for normality using the Shapiro–Wilk normality test and then grouped into parametric and non-parametric groups. The interaction between concentrations of analyzed membranes and used bacteria E.coli were analyzed using a one-way analysis of variance (STATISTICA, Stat Soft, Tulsa, OK, USA). The post hoc Tukey test was performed after each analysis. Statistical evaluation of differences in the individual bacterial strains between treatment groups was carried out using non-parametric statistics involving the Kruskal–Wallis test with multiple comparisons of mean ranks and the Mann–Whitney U test for individual groups. Differences were considered significant at *p* < 0.05, and all data are presented as mean ± standard error of the mean (SEM), (Table 1).

## 4. Conclusions

In this article, we have presented a novel class of antimicrobial agents: nanocomposite composed of spherical silica and silver ions located at the silica’s surface with the assumed distribution. After the analysis of their antimicrobial properties, we have shown that they induce very high oxidative stress in the cell, damaging and modifying bacterial DNA at the same time, creating oxidized guanines, cytosines, or adenines, which causes its very rapid destruction, leading to cell death. What is of particular importance in nosocomial infections of pathogenic bacterial strains. The influence of the silver ions concentration at the silica’s surface turned out to be crucial for the antibacterial properties of the investigated materials. During our research, we revealed that the lowest concentration of silver ions of the analyzed silica (sample SilS-COOAg 12) was the most effective and bactericidal of all the concentrations used. What was shown in the MIC and MBC tests carried out on model bacterial strains. This research raises hopes for introducing new materials based on functionalized silica as a substitute for silver nanoparticles into widespread use, which can significantly reduce the releasing silver and other metallic nanoparticles into the environment.

There is still a need to obtain more fundamental knowledge about the action of already synthesized and newly designed membranes treated by nanocomposite composed of spherical silica and silver ions located at the silica’s surface with the assumed distribution in vivo using high-throughput screening bioassays in bacterial systems for both types of Gram-stained bacteria. Another important task during the development of the practical use of various nanocomposites will be to understand their physiological effects under environmental conditions. In this regard, before the commercial use of these compounds, further studies on biostability, half-life, the most preferred mode of administration, and the potency of nanocomposites will be required.

## Figures and Tables

**Table 1:** Statistical analysis of all analyzed nanocomposites by MIC, MBC, and MBC/MIC. The sign * means statistical significance below 0.05.

Name of Sample	SilS-COOAg1	SilS-COOAg6	SilS-COOAg12	Type of Test
k12		*	*	MIC
R4		*	*	MIC
k12		*		MBC
R4		*		MBC
k12	*	*	*	MBC/MIC
R4	*	*	*	MBC/MIC

## Data Availability

The data presented in this study are available on request from the corresponding author.

## Abbreviations

The following abbreviations are used in this manuscript:

TEOS	tetraethyl orthosilicate
BNTES	butyronitrile triethoxysilane
ClTMS	trimethylsilyl chloride
Fpg	formamidopyrimidine-DNA glycosylase
MIC	minimal inhibitory concentration
MBC	minimum bactericidal concentration
LPS	lipopolysaccharides
